# Certificates for Hospital Life Governors

**Published:** 1913-10-04

**Authors:** Harry A. Bone

**Affiliations:** Secretary. The Miller General Hospital for South-East London, Greenwich Road, S.E.


*To the Editor of* The Hospital.

### Certificates for Hospital Life Governors.

*To the Editor of* The Hospital.

Sir,—I shall be greatly obliged if you will be so kind as to tell me whether you know of any hospital in London or the provinces which issues to its life governors or honorary life governors forms of certificate or of enrolment.

On two or three occasions recently we have been asked if we give such certificates, and it has occurred to us that some of the other hospitals may do so. Should you yourselves not know of any hospital which does this, perhaps one of your many readers may be able kindly to give us information on the point.—I am, Sir, yours faithfully,

Harry A. Bone, Secretary.

The Miller General Hospital for South-East London,
Greenwich Road, S.E.
October 1, 1913.

